# Effect of Genetically Reduced Maternal Myostatin on Late Gestation Maternal, Fetal, and Placental Metabolomes in Mice

**DOI:** 10.3390/metabo13060719

**Published:** 2023-06-01

**Authors:** Ruth Opoku, Jenna DeCata, Charlotte L. Phillips, Laura C. Schulz

**Affiliations:** 1 Division of Biological Sciences, University of Missouri, Columbia, MO 65211, USA; opokur@mail.missouri.edu (R.O.); jad5k7@umkc.edu (J.D.); 2 Department of Biochemistry, University of Missouri, Columbia, MO 65211, USA; phillipscl@missouri.edu; 3 Department of Obstetrics, Gynecology and Women’s Health, University of Missouri, Columbia, MO 65212, USA

**Keywords:** placenta, myostatin, intrauterine growth restriction, metabolism, vitamin C, fatty acid oxidation, polyamines, lysophospholipids

## Abstract

Myostatin (gene symbol: *Mstn*) is an autocrine and paracrine inhibitor of muscle growth. Pregnant mice with genetically reduced levels of myostatin give birth to offspring with greater adult muscle mass and bone biomechanical strength. However, maternal myostatin is not detectable in fetal circulations. Fetal growth is dependent on the maternal environment, and the provisioning of nutrients and growth factors by the placenta. Thus, this study examined the effect of reduced maternal myostatin on maternal and fetal serum metabolomes, as well as the placental metabolome. Fetal and maternal serum metabolomes were highly distinct, which is consistent with the role of the placenta in creating a specific fetal nutrient environment. There was no effect from myostatin on maternal glucose tolerance or fasting insulin. In comparisons between pregnant control and *Mstn*^+/−^ mice, there were more significantly different metabolite concentrations in fetal serum, at 50, than in the mother’s serum at 33, confirming the effect of maternal myostatin reduction on the fetal metabolic milieu. Polyamines, lysophospholipids, fatty acid oxidation, and vitamin C, in fetal serum, were all affected by maternal myostatin reduction.

## 1. Introduction

Intrauterine growth restriction (IUGR) is a pathological state where fetal growth is not able to meet its full potential. In clinical practice, IUGR may be diagnosed on the basis of fetal size < 10th percentile for gestational age, or by reduced or absent umbilical blood flow by Doppler ultrasound [1]. Pregnancy complications, such as preterm labor and stillbirth, and neonatal complications, including respiratory distress, hypoglycemia, and neurological impairment, are more common when IUGR is present [2]. The adult health of affected fetuses is also impaired by IUGR, with reductions in height, lean muscle mass, and bone strength, as well as increases in relative fat mass and insulin resistance [1,3]. Fetal genetic mutations can cause IUGR, which occurs in severe osteogenesis imperfecta [4], as can maternal undernutrition, although placental insufficiency is thought to be the most common cause [1,5,6,7]. 

Myostatin (gene symbol *Mstn*), or GDF8, is a member of the transforming growth factor beta (TGF-β) superfamily that acts in a paracrine fashion to reduce muscle growth [8]. Fetal musculoskeletal growth is inhibited, both by myostatin made by the developing fetus, and myostatin within the maternal circulation [9,10,11]. Lee [9] found that weights of the pectoralis, triceps, quadriceps, and gastrocnemius muscles in 10-week-old male *Mstn*^+/−^ offspring correlated with their mothers’ myostatin genotypes, with offspring of *Mstn*^−/−^ mothers having the largest muscle weights, and offspring of *Mstn*^+/+^ mothers having the smallest, while offspring of *Mstn*^+/−^ mothers were in-between [9]. Cross-fostering at birth did not alter the relationship between maternal myostatin genotype and offspring muscle weights, suggesting that it is maternal myostatin in the intrauterine environment rather than the postnatal lactational environment that regulates offspring muscle development [9]. Similarly, we have previously found that wildtype (WT) fetuses are larger when born to mouse mothers with genetically reduced myostatin (*Mstn*^+/−^) than when born to control mothers [12]. WT offspring from *Mstn*^+/−^ mothers also have enhanced bone biomechanical strength in adulthood, suggesting that maternal myostatin may be an important regulator of fetal bone, as well as muscle, development [12]. Increased bone biomechanical strength was also observed in *Col1a2*^+/−^ (osteogenesis imperfecta) fetuses, which have inherent bone fragility when born to *Mstn*^+/−^ mothers compared to control mothers [12]. The effect of reducing myostatin on adult bone biomechanics was recapitulated by transferring embryos at the morula stage into control or *Mstn*^+/−^ recipient mothers, showing that it is the intrauterine environment that confers greater bone strength [12]. Maternal myostatin influences fetal musculoskeletal phenotype without directly entering the fetal circulation. While *Mstn*^+/−^ mothers have approximately 30% lower serum myostatin concentrations than wildtype mothers, fetuses from the two maternal genotypes have identical serum myostatin concentrations, and myostatin null fetuses have no detectable serum myostatin, even when it is present in their mothers’ circulation [12].

As a result of these observations, we hypothesized that maternal myostatin may influence fetal musculoskeletal development by altering the metabolic environment that supports fetal growth. Maternal myostatin reduction may change the metabolism of the mother, and thus, the nutrients that are available for transport to the fetus. Alternatively, it may affect placental transport functions, meaning that nutrients from the maternal circulation reach the fetal circulation at different levels, or it may affect both transport and maternal metabolism. The goal of this study was to broadly characterize the metabolites present in the maternal and fetal serums, as well as placental tissue, from wildtype and *Mstn*^+/−^ mothers, using a metabolomics approach. Maternal metabolism was additionally characterized in myostatin deficient mothers by glucose tolerance testing and measurement of fasting insulin and serum triglyceride concentrations.

## 2. Materials and Methods

### 2.1. Animals

*Mstn^tm1Sj1^* mice, provided by S.J. Lee, contain a neo cassette instead of the C-terminal region of the *Mstn* gene and do not produce myostatin [13]. These mice were previously backcrossed into a C57Bl/6J background (Jackson Laboratories) [14]. For this study, both *Mstn*^+/−^ and WT control females were obtained from *Mstn^+/−^* x *Mstn*^+/+^ crosses. Genotypes were determined by PCR amplification using Terra PCR Direct Kit (Takara), with the following primer sets (Integrated DNA Technologies): (*Mstn* allele (forward: 5′GGATCGGCCATTGAACAAGATG3′ and reverse: 5′GAGCAAGGTGAGATGACAGGAG3′); wildtype allele (forward: 5′AGTCAAGGTGACAGACACACCCAA3′ and reverse: 5′TGGTGCACAAAGATGAGTATGCGGA3′)), generating 500 bp and 225 bp amplicons, respectively [15]. For both the glucose tolerance testing and metabolomics experiments, WT mothers were paired with *Mstn*^+/−^ sires (control cross) and WT sires were paired with *Mstn*^+/−^ mothers (experimental cross). The morning that a copulation plug was detected was considered gestation d0.5. 

### 2.2. Intraperitoneal Glucose Tolerance Testing (IPGTT)

Virgin non-pregnant WT (n = 8) and *Mstn*^+/−^ (n = 7) females and gestation d17.5 WT (n = 11) and *Mstn*^+/−^ (n = 8) females were not provided food from 07:00 to 12:00, with ad libitum access to water. Glucose tolerance testing was performed, according to the National Institutes of Health animal models of diabetic complications consortium protocol [16]. After 5 h fasting, mice were weighed, and a tail blood sample was taken for measurement of glucose and insulin. Then, 1 mg/mL glucose solution was injected intraperitoneally, and blood glucose concentrations were measured again by taking the mean from two ReliOn Prime glucometer readings (Walmart) at 15-, 30-, 60-, and 120-min post-injection. At the conclusion of testing, mice were euthanized by CO_2_ asphyxiation and cervical dislocation, and maternal blood was collected by cardiac puncture to determine serum triglyceride concentrations. Fetuses and placentas were weighed, and then, trunk blood was collected and pooled for each litter, to measure serum triglycerides. 

### 2.3. Insulin and Triglyceride Assays

Fasting insulin was measured with the Rat/Mouse Insulin ELISA kit (Millipore Sigma EZRMI13K) in 10 µL serum samples in duplicate, according to the manufacturer’s protocol, but with primary antibody incubation times extended to overnight. Briefly, 10 µL of matrix solution was added to blank, standard, and control wells, and all standards and samples were added in duplicate to the coated test plates and incubated with the detection antibody overnight at 4 °C. Wells were washed three times, incubated with enzyme solution, and then, washed again before addition of substrate solution. The stop solution was added, and absorbance was measured at both 450 nm and 590 nm in a Biotek plate spectrophotometer. A four-point standard curve was used to calculate sample concentrations by using Gen5 software. The mean intra-assay coefficient of variation was 6.7%. According to the manufacturer, the assay sensitivity was 0.2 ng/mL, and the range of the standard curve was 0.2–10 ng/mL.

Serum triglyceride concentrations were measured using the Triglyceride Assay Kit (Abcam ab65336), according to the manufacturer’s protocol for colorimetric (absorbance) assay. Briefly, glycerol standards or 2–5 µL of each serum sample were adjusted to a total volume of 50 µL in assay buffer. For each sample or standard, duplicate wells were incubated with Lipase or Triglyceride Assay buffer for 20 min to determine the total and free glycerol, respectively. Then, all wells were incubated in triglyceride enzyme mix and probe for 60 min before the absorbance was measured at 570 nm. Sample glycerol concentrations were determined from the standard curve. Triglyceride concentrations were determined by subtracting free glycerol from total glycerol, and then, adjusting for sample volume. The mean intra-assay coefficient of variation was 7.1%. According to the manufacturer, the assay sensitivity was 2 µM, and the range was 0–10 nmol.

### 2.4. Metabolomics

WT (n = 14) and *Mstn^+/−^* (n = 13) mothers were euthanized on day 17.5 of gestation and maternal serum, fetal serum, and placentas were collected. Fetal tails were collected for genotyping, as described above, and PCR sexing by *Rbm31x/y* amplification, as described previously [17]. One male, WT placenta was selected from each litter. Serum from all of the fetuses in each litter was combined to create one fetal serum pool for each mother. Eight litters from each maternal genotype provided a serum pool of sufficient volume for analysis. Metabolomic analysis was carried out by Metabolon Inc (Morrisville, NC, USA). Samples were prepared and extraction of metabolites was performed using ultra-high-performance liquid chromatography/tandem high resolution/accurate mass spectrometry in both positive and negative ion modes, using combination reverse phase and HILIC chromatography methods, as described previously [18]. Automated peak detection was used to determine the relative ions concentrations and matching of ion features to compound identities was conducted using Metabolon’s patented platform system software and a 4500+ named and 2750+ un-named compound reference library [19].

### 2.5. Statistical Analysis

All analyses, other than metabolomic profiles, were carried out using GraphPad Prism software. Maternal weights were compared between WT and *Mstn*^+/−^ mothers by Student’s *t*-test, with F-test to ensure equal variances. Maternal and fetal serum triglyceride concentrations and fetal and placental weights were analyzed by one-way ANOVA, with Bartlett’s test for equal variance. Glucose tolerance was compared by repeated measures ANOVA, with group and time as factors. Insulin concentrations showed significantly different variances between WT and *Mstn*^+/−^ mothers by F test, and therefore, medians were compared by Mann–Whitney test. For metabolomics, comparisons were made on log-transformed data using ArrayStudio/Jupyter Notebook software, while R. Welch’s two-sample t-tests were performed to identify biochemicals that differed between maternal and fetal serum and differed between WT and *Mstn*^+/−^ mothers within placentas, maternal serum, or fetal serum. Two-way ANOVA was used to identify significant interations between serum source (maternal vs. fetal) and maternal genotype. False discovery rate was estimated using q-value [20].

## 3. Results

### 3.1. Growth and Maternal Metabolic Assessment

**Figure 1:**
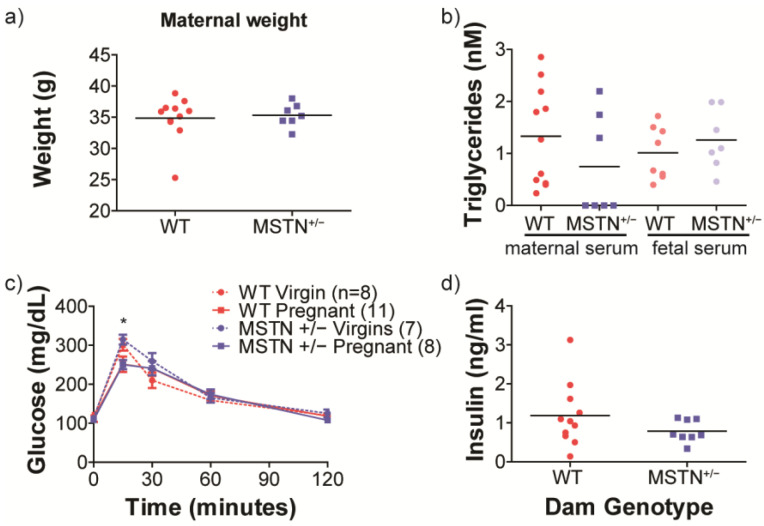
(**a**) Maternal pregnancy weights in control (WT) and *Mstn^+/−^* females. (**b**) Serum triglyceride concentrations in WT and *Mstn^+/−^* mothers and in serum pooled from all the fetuses in each litter, following IPGTT. (**c**) Intraperitoneal glucose tolerance testing in both pregnant and virgin control and *Mstn^+/−^* females. (**d**) Maternal fasting insulin concentrations in WT and *Mstn^+/−^* females prior to IPGTT. * *p* < 0.05 WT pregnant vs. *Mstn^+/−^* virgin at 15 min. (**a**,**b**,**d**) Each symbol represents data from an individual female, and the line represents the group means. (**c**) Each symbol represents the mean, and the bars represent SEM.

**Figure 2:**
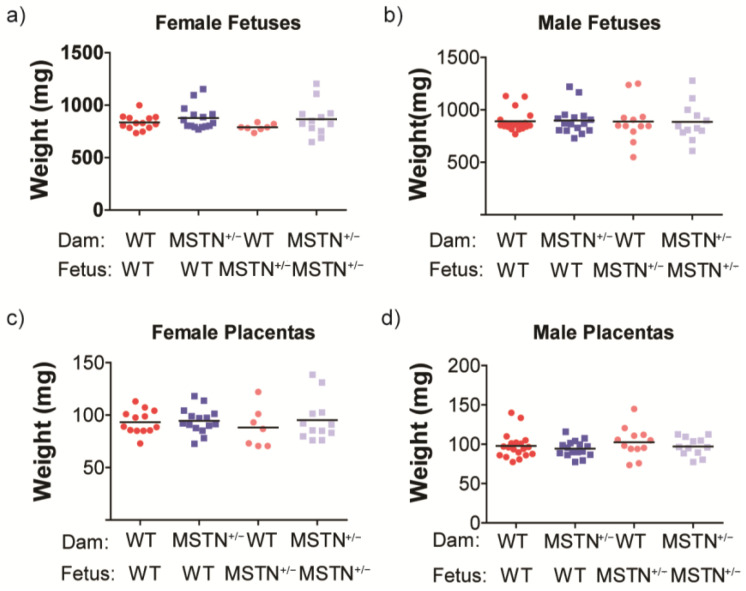
(**a**) Female and (**b**) male fetal weights on gestation d17.5, separated by maternal and fetal genotype. (**c**) Female and (**d**) male placental weights on gestation d17.5, separated by maternal and fetal genotype. There were no significant differences. Litters from females used in both the IPGTT and metabolomics experiments were used. Each symbol represents the mean weight of all fetuses of the same sex x fetal genotype combination within a single litter and the line represents the mean of all litters.

Maternal body weights in late pregnancy were not different between wildtype and *Mstn*^+/−^ females (Figure 1a). Maternal triglyceride concentrations were not significantly different between maternal genotypes in terminal blood samples taken from pregnant mothers or their fetuses. However, there was some indication of an interaction between maternal genotype and serum source, with maternal triglycerides tending to be lower, and fetal triglycerides tending to be higher in the *Mstn*^+/−^ pregnancies compared to controls (WT) (*p* = 0.15) (Figure 1b). Intraperitoneal glucose tolerance testing (IPGTT) was carried out in wildtype and *Mstn*^+/−^ females, both in the non-pregnant state and on gestation d17.5. Myostatin genotype had no effect overall on glucose tolerance (Figure 1c). However, there was a significant interaction between group and time, such that WT pregnant females had lower glucose than *Mstn*^+/−^ virgins at the 15 min time point. Fasting insulin measured in serum collected from pregnant mothers prior to the IPGTT did not differ according to maternal genotype, though there was significantly more variability (F test, *p* = 0.01) among WT mothers than among *Mstn*^+/−^ mothers (Figure 2d). 

Maternal, fetal, and placental weights were assessed in all pregnancies studied in both glucose tolerance testing and metabolomics analyses described below. There were no significant differences in fetal weight based on either maternal or fetal myostatin genotype (Figure 2). Litter sizes varied from 5 to 10 and did not differ significantly between WT (7.9 + 0.3) and *Mstn*^+/−^ (7.4 + 0.3) mothers.

### 3.2. Global Metabolomic Assessment

**Figure 3:**
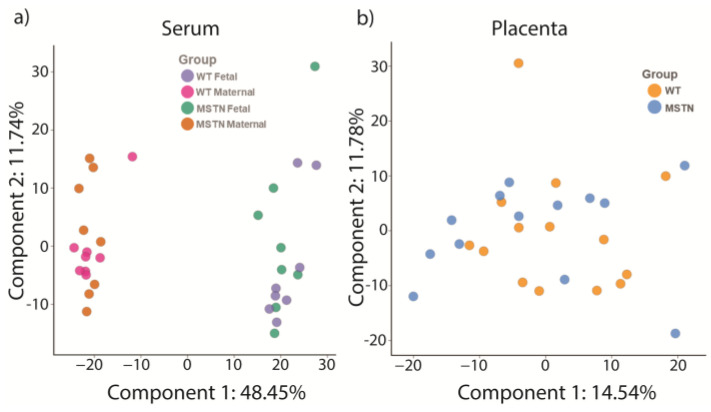
(**a**) Principal Component Analysis of metabolomic profiles of maternal and fetal sera in late pregnancy. (**b**) Principal Component Analysis of metabolomics profiles of wildtype, male placental tissue carried by WT and *Mstn^+/−^* mothers on gestation d17.5. Each symbol represents an individual serum or placental sample.

Metabolomic analysis was conducted on maternal serum from pregnant WT and *Mstn^+/−^* females, and in the pooled fetal serum from each of their litters. In order to control for fetal sex and fetal genotype, one WT male placenta from each litter was selected for tissue metabolic analysis. Overall, 917 metabolites were detected and identified in serum and 815 metabolites were identified and detected in placental tissue. The metabolomic analysis revealed a strong separation between the maternal and fetal serum metabolomes, which were easily distinguished by the Principal Components Analysis (Figure 3a). Accordingly, there were approximately 700 metabolites with statistically significant concentration differences between maternal and fetal sera, in both WT and *Mstn^+/−^* pregnancies (Table 1) and a clear separation between maternal and fetal samples in the Principal Component Analysis (Figure 3). Metabolite profiles did not differ as much by genotype as they did by maternal vs. fetal origin, with dozens rather than hundreds of differences. Maternal serum samples from WT mothers mostly clustered tightly, with *Mstn^+/−^* mothers (and one WT sample) distributing broadly in PCA component 2. There was no clear separation between the genotypes in the Principal Component Analysis of either fetal serum or placental samples (Table 1 and Figure 3). Nonetheless, there were statistically significant differences between WT and *Mstn^+/−^* metabolites in both the serum and placenta (Table 1). 

### 3.3. Maternal Sera Metabolite Differences Based on Maternal Myostatin Genotype

There were a total of 33 metabolites significantly up- or downregulated in the serum of pregnant *Mstn^+/−^* mothers compared to WT mothers, though none were significant after false discovery rate correction (q-value) (Table 2, Supplemental Table S1). Two of the three most significantly downregulated metabolites were oxidized forms of glutathione, a key cellular antioxidant, with the other most significantly downregulated metabolite being N-acetylated proline, which is found in acetylated peptides. The most significantly upregulated metabolite, 3-bromo-5-chloro-2,6-dihydroxybenzoic acid, is a salicylic acid compound. 

### 3.4. Fetal Sera Metabolite Differences Based on Maternal Myostatin Genotype

There were more metabolite differences in fetal serum from WT and *Mstn*^+/−^ mothers at 50 metabolites, than in the serum of the mothers themselves (Table 3, Supplemental Table S1). The most significantly upregulated metabolite, remaining significant after false discovery rate correction, was the polyamine metabolite N-acetylputrescine. It was 2.6-fold higher in fetal serum from *Mstn*^+/−^ mothers. N-acetylputrescine was overall the second-ranked biochemical for its ability to distinguish amongst the serum types in a random forest analysis (Supplemental Figure S1). 

### 3.5. Placental Metabolite Differences Based on Maternal Myostatin Genotype

Twenty-three metabolites were downregulated in placental tissue from *Mstn*^+/−^ mothers compared to WT mothers, whereas only four metabolites were upregulated (Table 4, Supplemental Table S2). The most significantly downregulated of these metabolites was alpha-tocopherol (vitamin E), reduced by 1.4-fold and the most significantly upregulated was 2-deoxyinosine, which was 1.5-fold higher in placentas from *Mstn*^+/−^ mothers. 

### 3.6. Lipid Metabolism

Of the 30 top-ranking biochemicals identified in the Biochemical Importance Plot, nearly half (14) were lipid metabolites (Supplemental Figure S1). Among the lipids that were found at different concentrations in WT and *Mstn^+/−^* sera, species related to fatty acid oxidation and lysophospholipids were particularly well represented. A number of medium and long-chain acylcarnitine species were differentially concentrated in either maternal or fetal serum, independent of maternal genotype, with acylcarnitine medium chain and monounsaturated fatty acids being higher in maternal serum and acylcarnitine polyunsaturated fatty acids being higher in fetal serum (Table 5). Additionally, several acylcarnitine fatty acids were found at higher concentrations in fetal blood from *Mstn^+/−^* mothers than in fetal blood from WT mothers (Table 5). There were no differences between *Mstn^+/−^* and WT mothers in maternal serum concentrations or placental concentrations of acylcarnitines (Table 5). 

There were also differences in the concentrations of several lysophopholipid types, with significant upregulation in the fetal serum from *Mstn^+/−^* mothers compared to fetuses from WT mothers, and some evidence of decreases in their placental tissue (Table 6).

### 3.7. Purine Metabolism Differences Based on Maternal Myostatin Genotype

Many purine metabolites were upregulated in fetal serum relative to maternal serum (Table 7 and Supplemental Table S1). Additionally, some purine compounds were regulated by maternal genotype. While 2-deoxyinosine, the product of the adenosine deaminase (ADA) enzyme, was upregulated in placentas from *Mstn^+/−^* mothers, adenosine, an ADA substrate, was significantly reduced in serum from their fetuses, although the placental concentration of deoxyadenosine (not shown) and adenosine were not significantly affected by maternal genotype. Fetal serum from *Mstn^+/−^* mothers also had significantly reduced levels of AMP, and some evidence of a similar reduction in its metabolite, IMP, which is generated by various isoforms of adenosine monophosphate deaminase. AMP can also be converted to adenosine by 5′-nucleotidase (*Nt5e*) [21]. There was a significant reduction in fetal serum guanine and 5′-GMP and maternal serum guanosine concentrations in *Mstn^+/−^* mothers.

### 3.8. Branched-Chain Amino Acids

Branched-chain amino acid metabolites isoleucine, isovalerate, and methylsuccinate were elevated in fetal serum from *Mstn^+/−^* mothers (Table 8). 

### 3.9. Vitamins 

In addition to the reduction in tocopherol in placental tissue from *Mstn^+/−^* mothers, there were slight reductions in vitamin A and vitamin B6 (Table 4). Vitamin C-related metabolites were found at different concentrations in the sera from *Mstn^+/−^* mothers. In WT mothers, ascorbate levels were 23-fold higher in fetal serum than in maternal serum (Table 4). In contrast, ascorbate was only 1.5-fold higher in fetal serum than in maternal serum of *Mstn^+/−^* mothers, such that ascorbate was 14-fold higher in the serum of the fetuses carried by the WT mothers compared to the serum of fetuses carried by *Mstn^+/−^* mothers. Dehydroascorbate was also significantly lower in fetal serum from *Mstn^+/−^* mothers vs. that of WT mothers, but only by 1.7-fold. Thus, the ratio of ascorbate to dehydroascorbate was 3.8 in WT fetuses but only 0.5 in fetuses from *Mstn^+/−^* mothers, suggesting that they may be more rapidly utilizing ascorbate. As mentioned above, GSSG was lower in maternal serum from *Mstn^+/−^* females (Table 2). One of the many redox reactions involving GSSG is the recycling of dehydroascorbate to ascorbate. Additionally, in maternal serum, though there were no differences in ascorbate or dehydroascorbate, there was a significant reduction in 2-O-methylascorbic acid, which is generated from ascorbate by catechol-O-methyltransfersase (COMT), an enzyme both widely expressed and abundant in the placenta [22,23] (Table 2).

## 4. Discussion

The largest metabolome differences observed here were those between fetal and maternal serum and were independent of maternal genotype. This reflects the critical role of the placenta in keeping the maternal and fetal circulations separate, acting not merely as a “sieve” or passive conduit for maternal serum components but actively enriching or blocking various components of maternal serum to maintain a unique fetal metabolic environment. Nonetheless, there were significant differences in maternal, fetal, and placental metabolomes based on maternal myostatin genotype. Strikingly, fetal serum metabolomes were more different between WT and *Mstn^+/−^* mothers than the maternal serum metabolomes. This was true even though the fetal genotypes were the same in WT and *Mstn^+/−^* mothers. WT females were mated to *Mstn^+/−^* sires, and vice versa, and fetal sera were pooled within each litter. Additionally, maternal myostatin does not enter the fetal circulation [12]. Thus, a slight reduction in maternal myostatin, acting in the maternal circulation, actually affects fetal metabolism more than it affects maternal metabolism. This is reinforced by our other assessments of maternal glucose metabolism; there were no differences in maternal glucose tolerance or fasting insulin concentrations.

The metabolite that most distinguished fetal serum from WT and *Mstn^+/−^* mothers was the polyamine N-acetylputrescine, while another polyamine metabolite, N-acetyl-cadaverine was elevated in placental tissue from *Mstn^+/−^* mothers. N-acetylarginine, a precursor of ornithine, which is subsequently converted to putrescine by ornithine decarboxylase (ODC1), was slightly, although significantly reduced in the maternal serum of *Mstn^+/−^* females (Table 2). Individuals with gain of function mutations in ODC1 have elevated N-acetylputrescine accompanied by N-acetylarginine deficiency, suggesting that myostatin reduction may be stimulating ODC1 activity in the uterus or placenta [24]. Putrescine promotes cell proliferation, protein synthesis, and mTOR phosphorylation in porcine placental cells [25]. ODC-null murine embryos die shortly after embryo implantation, with loss of the inner cell mass [26]. Similarly, pharmacological inhibition of ODC at various times between days 4 and 9, but not 1 and 3 or 10 and 18, resulted in the post-implantation embryonic loss, which could be rescued by putrescine replacement [27,28]. ODC1 expression in the mouse uterus normally peaks at gestation d8, [27,28]. ODC1 activation and putrescine treatment are associated with metabolic activation of blastocysts from embryonic diapause in both the mouse and the mink, [29,30]. Although the relationship between myostatin and ODC1 has not been studied, ODC1 does stimulate muscle growth [31,32]. Thus, ODC1 activity and putrescine production by either the uterus or placenta could plausibly be a mechanism for myostatin reduction to stimulate fetal metabolism and promote fetal growth. Importantly, however, here the polyamine metabolites were only measured near the end of gestation. Assessments of uterine and placental ODC1 activity and polyamine levels earlier across gestation in myostatin-deficient mothers would generate a fuller picture.

Medium and long-chain acylcarnitine fatty acids were increased in fetal serum from *Mstn^+/−^* mothers. Acylcarnitine fatty acids are created by the addition of carnitine to acyl-CoA conjugated fatty acids by CPT1, allowing transport into the mitochondrial matrix, and they accumulate when the supply of fatty acids exceeds their beta-oxidation by mitochondria [33]. Although beta-oxidation of fatty acids contributes significantly to placental energy generation [34], the placental content of these species did not differ between WT and *Mstn^+/−^* mothers. One possibility is that reduced fetal fatty acid oxidation may account for the higher fetal carnitine fatty acid concentrations. Fatty acids not used by fetuses can accumulate in the maternal circulation, as seen in mothers who carry fetuses with inborn errors in fatty acid oxidation [35]. Here, however, maternal acylcarnitine fatty acids were not different in serum from WT and *Mstn^+/−^* mothers. Alternatively, the elevated carnitine fatty acid levels in fetal serum may be a result of the placenta breaking down triglycerides to supply the fetus with higher levels of fatty acids for beta-oxidation in *Mstn^+/−^* mothers. This is somewhat supported by the serum triglyceride concentrations in *Mstn^+/−^* mothers, which were reduced, although not significantly. An increased supply of fatty acids for beta-oxidation would also be consistent with previous findings that myostatin knockout increased fatty acid oxidation enzyme levels in muscle in cattle [36] and in adipose tissue in mice [37]. 

Several lysophospholipids were significantly elevated in the fetal serum from *Mstn^+/−^* mothers compared to from WT mothers, with some evidence of decreases in the placentas from *Mstn^+/−^* mothers. These included glycerophosphatidic acid (GPA), glycerophosphorylcholine (GPC), glycerophosphoethalolamine (GPE), and glycerophosphatidylinositol (GPI) species. Lysophospholipids are small bioactive lipid molecules characterized by a single hydrophobic carbon chain and a polar head group attached to either a sphingoid base backbone, for the lysosphingolipids, or a glycerol backbone, for the lysoglycerophospholipids [38]. The lysolipid structure renders these lipids more hydrophilic, which makes them more versatile than other corresponding phospholipids. They act as extracellular mediators activating specific G-protein-coupled receptors (GPCRs), although some of them can also play a role in intracellular signal transduction [39]. Sphingosine-1-phosphate (S1P) and lysophosphatidic acid (LPA) have been characterized in the greatest detail up to this time, and are important in numerous biological functions, many potentially relevant to the effect of maternal myostatin on fetal growth. LPA receptors are expressed by osteoblasts and osteoclasts, and biomaterials containing LPA can enhance osteogenesis in vitro [40]. S1P is suggested to have numerous roles in the placenta, including regulation of trophoblast invasion and syncytialization [41,42]. Lysophospholipids are also components of the cell membrane and local mediators that regulate development, tissue regeneration, and homeostasis, and are precursors for lipid synthesis. Lysophosphatidylcholine (LPC), the most abundant lysophospholipid in blood, is transported by MFSD2, which also transports acylcarnitines, and serves as the receptor for syncytin-2 during human trophoblast syncytialization [38,43].

Multiple metabolomic differences point to a possible increase in adenosine deaminase activity in the placenta in mothers with reduced myostatin. The most upregulated metabolite in placental tissue from *Mstn^+/−^* mothers was 2-deoxyinosine, and a number of adenosine metabolites were downregulated in fetal serum from *Mstn^+/−^* mothers. Adenosine deaminase (ADA), which converts adenosine and deoxyadenosine to inosine and 2-deoxyinosine, is highly expressed in the placenta and is a key regulator of fetal adenosine concentrations [44]. Placental ADA is critical for placental development and fetal growth. While *Ada* knockout mice do not survive until birth, replacing ADA in just the placenta reduces fetal adenosine concentrations, alleviates fetal liver damage, and rescues the fetuses [44]. ADA supplementation also alleviated symptoms in a mouse model of preeclampsia and growth restriction based on angiotensin II overactivation. Combined placental and decidual *Ada* knockout results in neonatal mortality [45]. In humans, plasma adenosine is elevated in the cord blood of fetuses with pre-eclampsia and reduced uterine artery blood flow [46]. In these studies, reduced fetal serum adenosine is associated with better placental oxygenation and fetal growth. On the other hand, adenosine is also reduced in the plasma of growth-restricted fetal sheep, along with uric acid, suggesting that adenosine levels alone are not a clear indicator of fetal growth rates [47]. Additional studies are needed to test whether ADA is directly regulated by myostatin and whether this mediates any of the effects of reduced maternal myostatin on offspring outcomes.

In addition to adenosine, AMP, which can be metabolized to adenosine, was significantly reduced in fetal serum from *Mstn^+/−^* mothers. The ratio of AMP to ATP is critical for regulating the activity of the enzyme AMP kinase (AMPK), a nutrient sensor and master regulator of metabolism. Thus, one would predict lower AMPK activity in fetuses from *Mstn^+/−^* mothers, though this remains to be tested. In conditions of high AMP, AMPK is activated, triggering multiple energy-conserving and energy-generating cellular activities, including increased mitochondrial biogenesis, enhanced fatty acid oxidation, and inhibition of fatty acid synthesis [48]. Thus, it is possible that the seemingly reduced fatty acid oxidation observed in fetal serum from *Mstn^+/−^* mothers was a result of the lower AMP and AMPK levels. Elevated pAMPK was found in placentas from IUGR and high-altitude pregnancies and in the hypothalamus of rats that had experienced growth restriction [49,50,51]. Myostatin knockout has been shown to inhibit AMPK in mouse skeletal muscle [52] but myostatin treatment inhibited AMPK activity in cultured myoblasts. Whether suppression of AMPK activity might be mediating some of the effects of reduced maternal myostatin on fetal growth and development should be studied further. 

Guanine-containing-purines were also reduced in maternal (guanosine) and fetal (guanine, 5′-GMP) sera from *Mstn^+/−^* mothers, although the biological significance of these changes is not clear. While 5′GMP can be a metabolite of the signaling molecule cGMP, it is also a building block of nucleic acids. Guanylate cyclase and cGMP have been studied in the context of placental and uterine vasodilation in pregnancy, particularly in preeclamptic models, yet in fetal serum, no differences in cGMP were found in males with intrauterine growth restriction; thus, whether fetal cGMP concentrations play any role in regulating fetal growth is not understood [53,54]. 

Vitamin C (ascorbate) was significantly reduced in fetal serum from *Mstn^+/−^* mothers. This reduction could either result from reduced placental transport of ascorbate or increased consumption by the fetus; however, the reduced ratio of ascorbate to dehydroascorbate in fetuses from *Mstn^+/−^* mothers suggests that more ascorbate is being converted to dehydroascorbate. Ascorbate is a necessary cofactor in the synthesis of type I collagen, acting as a reducing agent in the hydroxylation of proline, which stabilizes the collagen triple helix [55]. We have previously shown an increased hydroxyproline content in the calvaria of 1–2-day-old WT neonates born to *Mstn^+/−^* mothers [12]. Thus, the improved bone architecture and biomechanical strength in the adult offspring of *Mstn^+/−^* mothers may result, at least in part, from increased rates of collagen synthesis beginning prenatally. These observations provide limited insight into the mechanisms through which maternal myostatin reduction stimulates fetal collagen synthesis, though increased placental ascorbate transport seems unlikely. The reduction in Vitamin A in placentas may be indicative of enhanced transport to the fetus. Vitamin E (alpha tocopherol) was reduced in both maternal serum and placental tissue from *Mstn^+/−^* mothers, but was not different in fetal serum, suggesting enhanced transport to the fetus. Vitamin A has mixed effects on bone development, with both excess and deficiency associated with reduced bone health [56]. Vitamin A concentrations in serum from pregnant women, but not cord blood, were shown to correlate positively with offspring bone mineral density in adults [57]. Vitamin E also does not appear to be required within the embryo, as embryonic knockout of its binding protein, TTPA, in mice, does not affect fetal growth, nor is it required for placental development after pregnancy at d13.5, meaning the significance of the reduction in vitamin E is unclear [58]. 

In contrast to our previous observations [12,59], there were no significant differences in fetal weight based on either fetal or maternal myostatin genotype. The lack of effect of fetal myostatin genotype was unexpected due to its well-established role in muscle development. However, the effects of heterozygosity are more subtle than complete knockout, and fine distinctions in the weights of mouse fetuses are difficult to detect. Whether maternal myostatin influences fetal weight and not just the musculoskeletal health of adult offspring is now unclear [9,12]. Body weights were also not larger in pregnant *Mstn^+/−^* females. McPherron and Lee found that females heterozygous for the myostatin mutation have larger muscles, but not significantly larger body weights, compared to WT mice at 2 months of age, though they were larger at 5 and 10 months of age [60]. Pregnant females in this study were 8–12 weeks of age.

Overall, metabolomic profiling showed a number of different biochemicals and biochemical classes that differ, particularly in fetal serum, in pregnancies from *Mstn^+/−^* mothers, and several of these provide plausible mechanisms for enhancing fetal musculoskeletal growth, either by altering energy supplies (carnitine fatty acids) or signaling molecules (lysophospholipids). Nearly all of the regulated metabolites in the placenta were found at lower concentrations (23 of 27), which is suggestive of nutrients being transferred rather than retained by the placenta. However, a limitation of these studies is an inability to determine whether differences in steady-state metabolite concentrations in maternal or fetal serum reflect differential transport or differential utilization by the mother or fetus. Thus, further experiments are needed to examine nutrient transporters and enzymes in the uterus and placenta, for example, by utilizing labeled tracers to directly assess transfers from mother to fetus. 

## Figures and Tables

**Table 1:** Summary of statistical comparisons of metabolites.

ANOVA Contrast	WT Fetal WT Maternal	*MSTN^+/−^* Fetal *MSTN^+/−^* Maternal	*MSTN^+/−^* Maternal WT Maternal	*MSTN^+/−^* Fetal WT Fetal	*MSTN^+/−^* Placenta WT Placenta
Total biochemicals *p* < 0.05	704	682	33	50	27
Biochemicals ↑|↓	393|311	412|270	17|16	40|10	4|23

↑ indicates upregulation and ↓ indicates downregulation.

**Table 2:** Metabolites altered in maternal plasma of pregnant *Mstn^+/−^* female compared to wildtype controls. N = 8 per group.

	*MSTN^+/−^* vs. WT		
Biochemical Name	Ratio	*p*-Value	q-Value
cysteine sulfinic acid	6.26	0.0367	0.9906
glu-gly-asn-val **	2.28	0.0456	0.9906
Erythronate *	2.16	0.0422	0.9906
3-bromo-5-chloro-2,6-dihydroxybenzoic acid *	2.09	0.0068	0.9906
2,3-dihydroxyisovalerate	1.90	0.0469	0.9906
heptanoate (7:0)	1.82	0.0283	0.9906
N,N,N-trimethyl-alanylproline betaine (TMAP)	1.68	0.0175	0.9906
lactosyl-N-palmitoyl-sphingosine (d18:1/16:0)	1.62	0.0216	0.9906
dodecanedioate (C12-DC)	1.50	0.0464	0.9906
3-acetylphenol sulfate	1.47	0.0104	0.9906
quinolinate	1.45	0.0103	0.9906
N-acetylkynurenine (2)	1.35	0.0355	0.9906
N-acetylthreonine	1.28	0.0225	0.9906
3,4-dihydroxybutyrate	1.28	0.0332	0.9906
2-O-methylascorbic acid	1.26	0.0113	0.9906
arabitol/xylitol	1.26	0.0479	0.9906
2-ketogulonate	1.24	0.0494	0.9906
N-acetylarginine	0.78	0.0294	0.9906
1-stearoyl-2-linoleoyl-GPE (18:0/18:2) *	0.71	0.0173	0.9906
alpha-tocopherol	0.62	0.0376	0.9906
1-myristoyl-2-arachidonoyl-GPC (14:0/20:4) *	0.61	0.0421	0.9906
1-(1-enyl-stearoyl)-2-oleoyl-GPE (P-18:0/18:1)	0.60	0.0058	0.9906
4-vinylcatechol sulfate	0.59	0.0334	0.9906
1-stearoyl-2-oleoyl-GPE (18:0/18:1)	0.57	0.0317	0.9906
N-acetylproline	0.53	0.0011	0.9853
N-(2-furoyl)glycine	0.50	0.0110	0.9906
pyrraline	0.48	0.0131	0.9906
N-acetylpyrraline	0.40	0.0155	0.9906
glutathione, oxidized (GSSG)	0.37	0.0091	0.9906
thromboxane B2	0.37	0.0313	0.9906
cysteine-glutathione disulfide	0.35	0.0074	0.9906
ribose 1-phosphate	0.33	0.0452	0.9906
cysteinylglycine disulfide *	0.15	0.0140	0.9906

* Identification not confirmed by an authentic chemical standard (not Metabolomic Standard Initiative Tier 1) but confidence in identity is high. ** identity not confirmed and confidence is moderate

**Table 3:** Metabolites altered in pooled plasma of fetuses carried by *Mstn^+/−^* mothers compared to wildtype controls at pregnancy d16.5. N = 8 L per group.

	*MSTN^+/−^* vs. WT		
Biochemical Name	Ratio	*p*-Value	q-Value
glycylleucine	3.85	0.0275	0.7158
1-arachidonoyl-GPA (20:4)	3.76	0.0479	0.8504
palmitoyl-arachidonoyl-glycerol (16:0/20:4) [1] *	3.15	0.0219	0.7158
oleoylcholine	2.71	0.0432	0.8504
N-acetylputrescine	2.63	1.9 × 10^−6^	0.0017
nicotinate ribonucleoside	2.29	0.0029	0.7158
1-arachidonylglycerol (20:4)	2.25	0.0003	0.1153
biopterin	2.20	0.0161	0.7158
caproate (6:0)	2.13	0.0050	0.7158
N-acetyl-3-methylhistidine *	1.97	0.0175	0.7158
2-docosahexaenoylglycerol (22:6) *	1.92	0.0163	0.7158
dihomo-linolenoylcarnitine (C20:3n3 or 6) *	1.78	0.0262	0.7158
isovalerate (i5:0)	1.73	0.0463	0.8504
cis-3,4-methyleneheptanoylcarnitine	1.67	0.0112	0.7158
N-acetyltyrosine	1.66	0.0236	0.7158
linolenoylcarnitine (C18:3) *	1.64	0.0147	0.7158
butyrate/isobutyrate (4:0)	1.64	0.0195	0.7158
N-acetylphenylalanine	1.61	0.0079	0.7158
methylsuccinate	1.59	0.0230	0.7158
S-(3-hydroxypropyl)mercapturic acid (HPMA)	1.57	0.0188	0.7158
N2,N5-diacetylornithine	1.56	0.0075	0.7158
3-acetylphenol sulfate	1.53	0.0418	0.8503
N-acetyltryptophan	1.51	0.0400	0.8503
mevalonate	1.48	0.0199	0.7158
succinoyltaurine	1.48	0.0255	0.7158
1-methyl-5-imidazolelactate	1.47	0.0349	0.7994
1-linoleoyl-GPC (18:2)	1.42	0.0259	0.7158
1-arachidonoyl-GPI (20:4) *	1.42	0.0263	0.7158
myristoyl dihydrosphingomyelin (d18:0/14:0) *	1.42	0.0410	0.8503
1-oleoyl-GPC (18:1)	1.41	0.0091	0.7158
N-acetylkynurenine (2)	1.41	0.0146	0.7158
1-stearoyl-GPC (18:0)	1.34	0.0281	0.7158
N2-acetyllysine	1.34	0.0315	0.7409
N-myristoyltaurine *	1.33	0.0146	0.7158
2-palmitoyl-GPC (16:0) *	1.32	0.0215	0.7158
N-acetylisoleucine	1.32	0.0299	0.7202
1-palmitoyl-GPC (16:0)	1.31	0.0105	0.7158
1-palmitoleoyl-GPC (16:1) *	1.29	0.0457	0.8504
3-methylhistidine	1.28	0.0297	0.7202
N6-acetyllysine	1.19	0.0147	0.7158
N1-methyladenosine	0.77	0.0276	0.7158
guanine	0.66	0.0139	0.7158
cytidine 5′-diphosphocholine	0.64	0.0388	0.8503
glycerol 3-phosphate	0.64	0.0406	0.8503
dehydroascorbate	0.58	0.0209	0.7158
gamma-glutamyl-2-aminobutyrate	0.47	0.0069	0.7158
guanosine 5′-monophosphate (5′-GMP)	0.33	0.0144	0.7158
adenosine	0.29	0.0127	0.7158
adenosine 5′-monophosphate (AMP)	0.12	0.0187	0.7158
ascorbate (vitamin C)	0.07	0.0482	0.8504

* Identification not confirmed by an authentic chemical standard (not Metabolomic Standard Initiative Tier 1).

**Table 4:** Metabolites altered in placental tissue of male fetuses carried by *Mstn^+/−^* mothers (N = 13) compared to wildtype (N = 14) at pregnancy d16.5.

	*MSTN^+/−^* vs. WT		
Biochemical Name	Ratio	*p*-Value	q-Value
4-acetamidobutanoate	1.53	0.0112	0.7972
2′-deoxyinosine	1.50	0.0011	0.7753
dihydroxyacetone phosphate (DHAP)	1.46	0.0153	0.7972
N-acetyl-cadaverine	1.42	0.0112	0.7972
1-palmitoyl-2-linoleoyl-GPC (16:0/18:2)	0.93	0.0434	0.7972
N2-acetyllysine	0.92	0.0472	0.7972
sphingomyelin (d18:1/20:1, d18:2/20:0) *	0.91	0.0468	0.7972
isoleucine	0.90	0.0300	0.7972
1-palmitoleoyl-2-linoleoyl-GPC (16:1/18:2) *	0.90	0.0331	0.7972
alanine	0.90	0.0419	0.7972
myo-inositol	0.89	0.0267	0.7972
fumarate	0.89	0.0349	0.7972
1-palmitoyl-2-palmitoleoyl-GPC (16:0/16:1) *	0.88	0.0464	0.7972
S-adenosylhomocysteine (SAH)	0.87	0.0267	0.7972
alpha-ketoglutarate	0.86	0.0194	0.7972
retinol (vitamin A)	0.85	0.0271	0.7972
glycerophosphorylcholine (GPC)	0.83	0.0490	0.7972
3-methylcytidine	0.80	0.0170	0.7972
pyridoxamine phosphate (vitamin B6)	0.79	0.0048	0.7972
3-aminoisobutyrate	0.77	0.0197	0.7972
1-palmitoyl-2-oleoyl-GPS (16:0/18:1)	0.74	0.0110	0.7972
alpha-tocopherol	0.73	0.0021	0.7866
gluconate	0.71	0.0443	0.7972
pyrraline	0.69	0.0048	0.7972
phytosphingosine	0.66	0.0477	0.7972
genistein	0.64	0.0314	0.7972
taurocholate	0.61	0.0437	0.7972

* Identification not confirmed by an authentic chemical standard (not Metabolomic Standard Initiative Tier 1).

**Table 5:** Maternal genotype-based differences in fatty acid beta-oxidation metabolites.

Pathway	Biochemical Name	Ratio				
		WT Fetal WT Maternal	*MSTN^+/−^* Fetal *MSTN^+/−^* Maternal	*MSTN^+/−^* Maternal WT Maternal	*MSTN^+/−^* Fetal WT Fetal	*MSTN^+/−^* Placenta WT Placenta
Acylcarnitine Medium Chain	cis-3,4-methylene heptanoylcarnitine	0.39 *	0.66 *	0.99	1.67 *	1.10
Acylcarnitine Monounsaturated	cis-4-decenoyl carnitine (C10:1)	0.21 *	0.26 *	1.07	**1.36** **†**	1.02
	Myristoleoyl carnitine (C14:1) *	0.30 *	0.35 *	1.14	**1.33** **†**	1.09
Acylcarnitine Polyunsaturated	Linolenoyl carnitine (C18:3) *	0.43 *	0.65 *	1.08	1.64 *	1.05
	dihomo-linoleoyl carnitine (C20:2) *	1.31	1.76 *	1.18	**1.58** **†**	1.0
	Arachidonoyl carnitine (C20:4)	0.79	1.06	1.02	**1.38** **†**	0.98
	dihomo-linolenoyl carnitine (C20:3n3 or 6) *	1.00	1.76 *	1.02	1.78 *	1.07
	docosapentaenoylcarnitine (C22:5n3) *	1.94 *	2.90 *	1.09	**1.62** **†**	1.00
	docosahexaenoylcarnitine (C22:6) *	0.63 *	0.93	1.02	**1.51** **†**	1.00
Carnitine	Carnitine	1.02	1.12	0.97	1.06	1.01

Ratios in **bold** are significant. *: *p* < 0.05, †: *p* > 0.05, <0.1, * identification not confirmed by an authentic chemical standard (not Metabolomic Standard Initiative Tier 1).

**Table 6:** Maternal genotype-based differences in lysophospholipid compounds.

Pathway	Biochemical Name	Ratio				
		WT Fetal WT Maternal	*MSTN^+/−^* Fetal *MSTN^+/−^* Maternal	*MSTN^+/−^* Maternal WT Maternal	*MSTN^+/−^* Fetal WT Fetal	*MSTN^+/−^* Placenta WT Placenta
Lysophospholipids	1-linoleoyl-GPA (18:2) *	0.28 *	0.59	1.47	**3.11 †**	ND
	1-arachidonoyl-GPA (20:4)	0.57	1.19	1.80	3.76 *	ND
	1-palmitoyl-GPC (16:0)	0.39 *	0.5 *	1.02	1.31 *	0.98
	2-palmitoyl-GPC (16:0) *	0.24 *	0.33 *	0.98	1.32 *	0.94
	1-palmitoleoyl-GPC (16:1) *	1.74 *	2.31 *	0.97	1.29 *	0.88
	1-stearoyl-GPC (18:0)	0.27 *	0.38 *	0.98	1.34 *	1.01
	1-oleoyl-GPC (18:1)	1.11	1.51 *	1.04	1.41 *	0.95
	1-linoleoyl-GPC (18:2)	0.38 *	0.54 *	0.98	1.42 *	0.92
	1-arachidonoyl-GPC (20:4n6) *	0.83	1.08	1.01	**1.33 †**	0.83
	2-stearoyl-GPE (18:0) *	0.28 *	0.38 *	**0.79 †**	1.04	0.84
	1-oleoyl-GPE (18:1)	0.94	1.63 *	0.64	1.11	0.98
	1-linoleoyl-GPE (18:2) *	0.34 *	0.5 *	0.85	1.27	**0.82 †**
	1-arachidonoyl-GPE (20:4n6) *	0.65 *	0.75	0.99	1.16	**0.86 †**
	1-oleoyl-GPS (18:1)	ND	ND	ND	ND	**0.76 †**
	1-linoleoyl-GPS (18:2) *	ND	ND	ND	ND	**0.78 †**
	1-oleoyl-GPG (18:1) *	12.93 *	9.59 *	1.61	1.19	**0.82 †**
	1-linoleoyl-GPG (18:2) *	0.98	1.04	1.23	**1.3 †**	0.84
	1-arachidonoyl-GPI (20:4) *	0.22 *	0.26 *	1.19	1.42 *	0.88

Ratios in **bold** are significant. *: *p* < 0.05, †: *p* > 0.05, <0.1, * identification not confirmed by an authentic chemical standard (not Metabolomic Standard Initiative Tier 1). ND: not different.

**Table 7:** Maternal genotype-based differences in purine metabolites.

Pathway	Biochemical Name	Ratio				
		WT Fetal WT Maternal	*MSTN^+/−^* Fetal *MSTN^+/−^* Maternal	*MSTN^+/−^* Maternal WT Maternal	*MSTN^+/−^* Fetal WT Fetal	*MSTN^+/−^* Placenta WT Placenta
Purine Metabolism, (Hypo)Xanthine/Inosine containing	2′-deoxyinosine	ND	ND	ND	ND	1.5 *
	inosine 5′-monophosphate (IMP)	15.16 *	2.00	1.00	**0.13 †**	ND
	N1-methylinosine	8.01 *	7.15 *	**1.26 †**	1.12	1.10
	allantoic acid	0.41 *	0.59 *	1.11	**1.61 †**	ND
Purine Metabolism, Adenine containing	adenosine 5′-monophosphate (AMP)	68.59 *	8.83 *	0.93	0.12 *	1.47
	adenosine 3′-monophosphate (3′-AMP)	**6.95 †**	5.8 *	0.78	0.66	**1.47 †**
	adenosine 2′-monophosphate (2′-AMP)	8.27	18.52 *	0.22	0.50	**1.85 †**
	adenosine	11.27 *	4.38 *	0.75	0.29 *	0.84
	N1-methyladenosine	3.04 *	2.32 *	1.00	0.77 *	**0.78 †**
	N6-succinyladenosine	**30.35**	16.6 *	1.22	**0.67 †**	0.92
Purine Metabolism, Guanine containing	guanosine 5′-monophosphate (5′-GMP)	57.36 *	36.38 *	0.52	0.33 *	0.96
	guanosine	53.73 *	132.89 *	**0.38 †**	0.95	0.95
	guanine	22.04 *	14.46 *	1.00	0.66 *	0.89

Ratios in **bold** are significant. *: *p* < 0.05, †: *p* > 0.05, <0.1, * identification not confirmed by an authentic chemical standard (not Metabolomic Standard Initiative Tier 1). ND: not different.

**Table 8:** Differences in branched-chain amino acid metabolites.

Pathway	Biochemical Name	Ratio				
		WT Fetal WT Maternal	*MSTN^+/−^* Fetal *MSTN^+/−^* Maternal	*MSTN^+/−^* Maternal WT Maternal	*MSTN^+/−^* Fetal WT Fetal	*MSTN^+/−^* Placenta WT Placenta
Leucine, isoleucine, and valine metabolisms	N-acetylleucine	0.63 *	0.89	0.91	1.28	1.08
	isovalerate (i5:0)	0.83	1.01	1.42	1.73 *	ND
	isovalerylcarnitine (C5)	2.58 *	3.96 *	0.85	1.30	**0.91 †**
	isoleucine	1.17	1.15	0.99	0.97	0.9 *
	N-acetylisoleucine	0.78 *	1.11	0.92	1.32 *	0.96
	2-methylbutyrylglycine	0.29 *	0.27 *	1.57	1.46	**1.68 †**
	methylsuccinate	0.69 *	0.81	1.36	1.59 *	1.08

Ratios in **bold** are significant. *: *p* < 0.05, †: *p* > 0.05, <0.1, * identification not confirmed by an authentic chemical standard (not Metabolomic Standard Initiative Tier 1). ND: not different.

## Data Availability

Full metabolomics data are openly available in the Metabolights repository at https://www.ebi.ac.uk/metabolights/MTBLS3345 study number MTBLS3345. Other data are contained within the article.

## Supplementary Materials

The following supporting information can be downloaded at: https://www.mdpi.com/article/10.3390/metabo13060719/s1. Figure S1. Random forest classification using named metabolites in mice groups: WT Fetal, WT Maternal, MSTN Fetal, and MSTN Maternal provided a predictive accuracy of 44%; Table S1. Effect of maternal myostatin on placental nutrient transfer (2nd serum); Table S2. Effect of maternal myostatin on placental nutrient transfer–placenta merged set.
