# Supplementary figures and images for: Effect of Genetically Reduced Maternal Myostatin on Late Gestation Maternal, Fetal, and Placental Metabolomes in Mice
Source: Metabolites. 2023 Jun 1;13(6):719. doi: 10.3390/metabo13060719 (PMC10302353; doi:10.3390/metabo13060719)

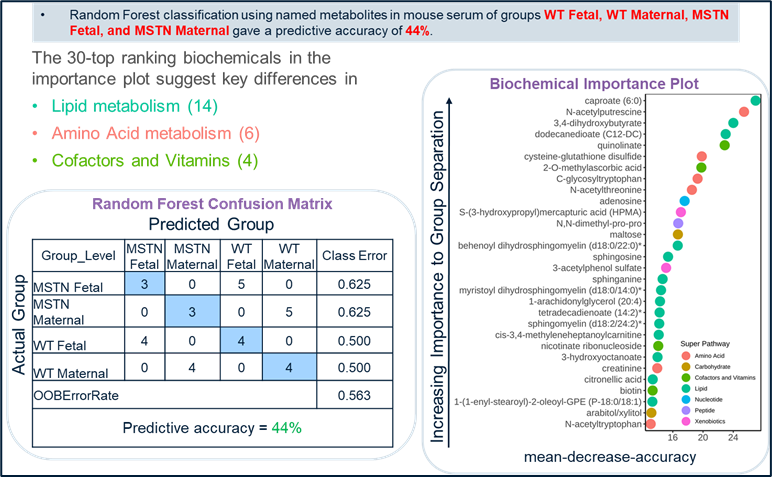

Supplement: Supplementary file 1 [file metabolites-13-00719-s001.zip › Suppl Fig 1 Random Forest.png]
